# Effect of Functional Training on Physical Fitness Among Athletes: A Systematic Review

**DOI:** 10.3389/fphys.2021.738878

**Published:** 2021-09-06

**Authors:** Wensheng Xiao, Kim Geok Soh, Mohd Rozilee Wazir Norjali Wazir, Othman Talib, Xiaorong Bai, Te Bu, He Sun, Stevo Popovic, Bojan Masanovic, Jovan Gardasevic

**Affiliations:** ^1^Department of Sports Studies, Faculty of Educational Studies, Universiti Putra Malaysia, Seri Kembangan, Malaysia; ^2^Department of Sports Studies, Faculty of Education Studies, Universiti Putra Malaysia, Seri Kembangan, Malaysia; ^3^Department of Science and Technical Education, Faculty of Educational Studies, Universiti Putra Malaysia, Seri Kembangan, Malaysia; ^4^Department of Sports Studies, Faculty of Education Studies, Hunan Normal University, Changsha, China; ^5^Faculty for Sport and Physical Education, University of Montenegro, Podgorica, Montenegro; ^6^Montenegrin Sports Academy (MSA), Podgorica, Montenegro; ^7^Montenegrosport, Podgorica, Montenegro

**Keywords:** flexibility, muscular endurance, body composition, balance, speed

## Abstract

There is evidence that functional training is beneficial for the overall physical fitness of athletes. However, there is a lack of a systematic review focused on the effects of functional training on athletes' physical fitness. Thus, the aimed of the present review is to clarify the effects of functional training on physical fitness among athletes. In accordance with the Preferred Reporting Items for Systematic Reviews and Meta-Analyzes (PRISMA) *Statement* guidelines, the systematic search of PubMed, SCOPUS, EBSCOhost (SPORTDiscus), and CINAHL Plus databases was undertaken on *the 2nd November* 2020 to identify the reported studies, using a combination of keywords related to functional training, physical fitness, *and* athletes. *From the 145 studies*, only nine articles met all eligibility criteria and were included in the systematic review. The assessment was performed on the Pedro scale, and the quality of the study included in the nine studies was fair (ranging from 3 to 4). The results showed that speed (*n* = 6) was the *aspect of physical fitness* studied in functional training interventions, followed by muscular strength (*n* = 5), power (*n* = 4), balance (*n* = 3), body composition (*n* = 3), agility (*n* = 3), flexibility (n = 1) and muscular endurance (*n* = 1). Existing evidence concludes that functional training significantly impacts speed, muscular strength, power, balance, and agility. *Furthermore, there are still limit numbers of evidence showing effect of* functional training on flexibility and muscular endurance. In contrast, no significant improvement was found in body composition where functional training was conducted.

**Systematic Review Registration:**https://www.crd.york.ac.uk/prospero, identifier: CRD4202123092.

## Introduction

Athletes' successful performance is usually attributed to the unique combination of talent and physical fitness, technical, tactical, and psychological qualities (Smith, 2003). Among those criteria, physical fitness is considered the most critical quality to determine athletes' competitive ability (Gabbett et al., 2007). Excellent physical fitness is essential for improving the athletes' technical and tactical level and performance and is the basic requirement for competing athletes under high-intensity training (Chunlei, 2016). The loss of an athlete's physical fitness component can jeopardize this ability and lead to sports injuries (Dengguang and Yang, 2007a). For example, in tennis players, *decreased muscle strength and postural control limits* the ability to start quickly and change direction, which further hinders their ability to hit the ball effectively and maintain a stable body, and also increased the likelihood of sports injuries (Kovacs, 2006).

A substantial number of publications proved a significant positive correlation between physical fitness components and exercise training intervention. *The American College of Sports Medicine guidelines support the use of traditional resistance training, traditional resistance training enhances physical fitness performance by gradually increasing exercise load during the training process (Feito et al.*, *2018**). However, the training specificity literature has shown that the benefits of traditional resistance training for improving physical fitness is rarely transferred to sports performance (Li et al.*, *2019**; Li*, *2021**). Most of the traditional resistance training methods are not multi-articular and multiplanar; these aspects seem fundamental for eliciting greater sports performance (Fernandez-Fernandez et al.*, *2016**; Santos-rosa et al.*, *2020**). On the other hand*, a new exercise training method that has recently received much attention to developing athletes' physical fitness is functional training (Feito et al., 2018). Several studies have confirmed that functional training can enhance speed (Tomljanović et al., 2011; Sander et al., 2013; Alonso-Fernández et al., 2017; Yildiz et al., 2019; Baron et al., 2020; Keiner et al., 2020), muscular strength (Oliver and Brezzo, 2009; Tomljanović et al., 2011; Elbadry, 2014; Cherepov and Shaikhetdinov, 2016; Keiner et al., 2020), power (Tomljanović et al., 2011; Alonso-Fernández et al., 2017; Yildiz et al., 2019; Keiner et al., 2020), balance (Oliver and Brezzo, 2009; Elbadry, 2014; Yildiz et al., 2019), body composition (Oliver and Brezzo, 2009; Tomljanović et al., 2011; Alonso-Fernández et al., 2017), agility (Tomljanović et al., 2011; Cherepov and Shaikhetdinov, 2016; Yildiz et al., 2019), flexibility (Yildiz et al., 2019) and muscular endurance (Oliver and Brezzo, 2009). Additionally, other research has discovered positive effects of functional training on physical fitness in football players (Oliver and Brezzo, 2009; Sander et al., 2013; Baron et al., 2020; Keiner et al., 2020), handball players (Elbadry, 2014; Alonso-Fernández et al., 2017), martial artists (Cherepov and Shaikhetdinov, 2016), tennis players (Yildiz et al., 2019) and volleyball players (Oliver and Brezzo, 2009). Despite the significance of functional training for improving the physical fitness components among athletes, there is no publication that summarized crucial information on the impacts of functional training protocols on physical fitness among athletes.

Conceptually, functional training refers to the training of partial chains and connections in the human motion chain that involves completing specific target actions, including multi-dimensional motion trajectory acceleration, deceleration, and stability training activities that meet the characteristics of particular target actions (Cook, 2012). The action mode of functional training involves acceleration, deceleration and stability of multiple joints and planes. The action mode determines the broad participation and effective pertinence of functional training (National Academy of Sports Medicine, 2001). Moreover, Boyle believes that the essence of functional training is purposeful training. It is a multi-plane exercise in stable control and weight-bearing. It is a series of exercises that involve balance and proprioception and are supported by body parts (Boyle, 2016). Therefore, functional training differs from traditional resistance training; it can be any exercise performed to enhance a specific movement or activity (Pacheco et al., 2013). With a definition this broad, the literature on functional training has incorporated various exercise programs with varying designs and focuses. The principle of functional training is the specificity of training, which means that training in a specific activity is the best way to maximize the performance in that particular area (Hawley, 2008). In other words, the closer the training is to the desired outcome (i.e., a specific task or performance criterion), the better the result will be. For example, when the functional training program includes the element of strength training, the training improves the outcome of muscle strength (Skelton et al., 1995; Alexander et al., 2001; Giné-Garriga et al., 2010). The results presented by the different studies on functional training effects on physical fitness components among athletes are encouraging, but limited scientific information is available to determine its possible benefits on the different *physical fitness components* of performance. Therefore, this systemtic review aimed of the present review is to clarify the effects of functional training on physical fitness among athletes.

## Methods

### Protocol and Registration

The data selection, collection and analysis of this review were performed following the Preferred Reporting Items for Systematic Reviews and Meta-Analyses (PRISMA) guidelines (Moher et al., 2009) and were prospectively registered on the International Prospective Register of Systematic Reviews; https://www.crd.york.ac.uk/prospero, CRD4202123092.

### Search Strategy

The literature search was undertaken in four international databases: the SCOUPS, PubMed, EBSCOhost (SPORTDiscus), and CINAHL Plus. The search was conducted on *the 2th November*, 2020. In each database, a search was conducted by title, taking a predefined combination of keywords: (“functional training” OR “functional exercise” OR “functional skill^*^” OR “functional task training” OR “therapeutic exercise”) AND (“physical fitness” OR “physical endurance” OR “cardiorespiratory fitness” OR “physical conditioning” OR “skill-related fitness” OR “skill related fitness” OR “skill related physical” OR “skill-related physical” OR “skill related physical fitness” OR “skill-related physical fitness” OR “fitness, physical” OR “speed” OR “power” OR “reaction time” OR “agility” OR “balance” OR “coordination” OR “health related physical fitness” OR “health related physical” OR “health related fitness” OR “health-related physical” OR “health-related fitness” OR “health-related physical fitness” OR “aerobic endurance” OR “muscular strength” OR “muscular endurance” OR “body composition” OR “flexibility”) AND (“player^*^” OR “athlete^*^” OR “sportsman^*^” OR “sportswoman^*^” OR “sportsperson^*^” OR “Jock^*^”). We also explored other relevant articles in the reference lists of the studies included in the review and examined the reference lists of previous related reviews. All titles were manually searched for potential inclusion. Reference lists of retrieved papers, authors' names, and review articles were retrieved manually for additional relevant citations.

### Eligibility Criteria

We used the PICOS (population, intervention, comparison, outcome, study designs) criteria as the inclusion criteria, is presented in Table 1. Only records presenting functional training on aspect of physical fitness of athletes were included. Thus, studies were included if they met the following criteria: (1) A full text, peer-reviewed study published in English, describing the use of athletes (male and female) to explore the effects of functional training interventions on physical fitness, randomized controlled trial (RCT), non-randomized controlled trial (Non-RCT) with two or more groups, and single-group trials with pretest and post-test design; (2) In this study, only included studies on planned and organized functional training intervention to improve or maintain physical fitness. Notably, studies using functional training or combinations of functional training and other exercise training interventions (e.g., resistance training) were also included from this review; (3) Investigate the effects of functional training on physical fitness among athletes and assess at least one physical fitness component outcome; (4) There were no restrictions on the sample size, study location, and intervention time for the included studies.

**Table 1:** Inclusion criteria according to the PICOS conditions.

**Items**	**Detailed inclusion criteria**
Population	Athletes (male/female)
Intervention	Functional training
Comparison	Two or more groups and single-group trials
Outcome	Physical fitness (speed, power, reaction time, agility, balance, coordination, aerobic endurance, muscular strength, muscular endurance, body composition, flexibility)
Study designs	RCT or Non-RCT

Studies were excluded if they met several exclusion criteria: (1) Studies that combined functional training interventions with additional non-exercise training (e.g., psychological interventions) and interventions including unsupervised training courses were not included in the study; (2) Studies published articles, meeting abstracts, case reports, and short communications in languages other than English were excluded; and (3) Observational studies and interventions focusing solely on counseling for functional training implementation were excluded.

### Study Selection

The retrieved studies were imported into Mendeley reference management software to remove any duplicates. Firstly, the search strategies were assisted by an experience librarian. Secondly, two independent reviewers (Xiao, Bai) screened the titles and abstracts of all the identified articles in the initial screening phase to identify relevant studies. Irrelevant materials were removed from the database before assessing all other titles and abstracts using our predetermined inclusion and exclusion criteria. Articles that remain at the end and enter a qualitative synthesis must have the whole text, and the whole text must be read. Items for which the full text is not available are dropped. If there were any disagreements, a third reviewer (Soh) was consulted until a consensus was achieved.

### Data Extraction and Quality Assessment

After the data search was complete, data were obtained from eligible studies in a predetermined extraction form [Including, (1) Author, title, publication year; (2) Research design; (3) Sample size, control group; (4) Participant characteristics (age, gender, etc.); (5) Intervention features (type, length, and frequency); (6) Measures index, and (7) Research outcomes]. One author abstracted information into the standard form and the other author checked it.

The PEDro scale (www.pedro.org.au) has been proven to be a useful measure of the quality of experimental methodology in developing a systematic review, and has good validity and reliability (Lima et al., 2013). The PEDro scale is designed to evaluate the four fundamental methodological aspects of a study, such as random process, blind technique, group comparison and data analysis. The assessment of the 11 items in the PEDro scale was performed by two well-trained, independent raters using a yes (1 point) or No (0 points) response rating scale, and disagreements were resolved by a third rater. However, the eligibility criteria were not considered in the total score since this was related to external validity. The total PEDro score ranges from 0 to 10 points, and higher scores reflect a better methodological quality. The higher the PEDro score, the higher the quality of the corresponding method. Studies scoring 8 to 10 were considered to be methodologically excellent in quality, those ranging from 5 to 7 to be good in quality, while a score between 3 and 4 is fair in quality, and those scoring below 3 to be poor in quality (Foley et al., 2003). The judgment of overall scientifific evidence was based on number, methodological quality and consistency of outcomes of the studies in three levels of evidence: (1) strong evidence, provided by generally consistent findings in multiple (≥2) number and results studies, (2) moderate evidence, when only one study is available or findings are inconsistent in multiple (≥2) studies, (5) no evidence, when no case-control studies are found.

## Results

The search results were screened and read by formulating literature inclusion and exclusion criteria. This systematic review contains nine articles involving RCT and Non-RCT on the effects of functional training on physical fitness among athletes. They were published between the years of 2009–2020. In Table 2, the studies' characteristics are presented.

**Table 2:** Characteristics of the studies examined in the present review.

**Study**	**Design**	**Type of athletes**	**Population characteristics**	**Interventions**	**Type of exercise training**	**Measures index**	**Outcomes**
Oliver and Brezzo (2009)	Pre-post test	Collegiate athletes (volleyball and soccer players)	Sex: F, TB: NR, EG1 = 15, Age: 19.9 ± 1.8yr., WT: 71.8 ± 8.5kg, ht.: 174.5 ± 11.9cm, BMI: NR, CG = 11, Age: 18.5 ± 0.5yr., WT: 63.3 ± 6.7kg, ht.: 166.4 ± 5.6cm, BMI: NR	Freq.: 4 times/week, time: 10 min, length: 13 weeks	Functional balance training group (EG1), control group (CG)	Body composition (WT, BF, BMI), strength (quadra-ped: left, right; single leg squat (left, right), muscular endurance (sit up), balance (biodex balance test: left, right)	Single leg squat (right, left)↑, sit up↑, BMI↔, WT↔, BF↔, quadra-ped (left, right)↔, Biodex (right, left)↔
Tomljanović et al. (2011)	Pre-post test	Moderately trained athlete	Sex: M, TB≥146 months, Age: 22–25yr., EG1 = 11, WT: 78.89 ± 12.32, ht.: 179.69 ± 6.39, CG = 12, WT: 82.42 ± 12.92, ht.: 185.00 ± 10.58, BMI: NR	Freq.: 3 times/week, time: NR, Length: 5 weeks	Functional training (EG1), traditional resistance training (CG)	Agility (5-10-5 test, HEX), power (CMJ: AT, PEAKPWR, JH, GCT), strength (SMB, LMB), speed (10 m, 20 m, 10–20 m), body composition (WT, ht., BF%, BF, LBM, H2o)	HEX↑, SMB↑, JH↑, PEAKPWR↑, GCT↑, LMB↔, AT↔, 5–10–5↔, 10m↔, 20 m↔, 10–20 m↔, WT↔, ht.↔, BF%↔, BF↔, LBM↔, H2o(I)↔
Sander et al. (2013)	Pre-post test	Elite youth soccer players	EG1 = 65, EG2 = 56, Sex: NR, TB≥146 months, mean age: 15.1yr., Mean ht.: 170.9, Mean WT: 62.3, BMI: NR	Freq.: NR, time: NR, Length: 8 days	Completed the NWP first and the WPS 4 days later (EG1), completed the WPS first and the NWP 4 days later (EG2)	Speed (linear sprint: 5 m, 10 m, 15 m, 20 m, 25 m, 30 m, and CDS:5 m left and right, 10m left and right)	5 m↑, 10 m↑, 15 m↑, 20 m↑, 25 m↑, 30 m↑, 5 m left↔, 5 m right↑, 10 m left↑, 5 m right↑
Elbadry (2014)	Pre-post test	Young handball players	EG1: n = 10, Sex: F, TB: 3 ± 0.7yr., Age: 13 ± 1.5yr, WT: 44 ± 2.7, ht.: 147 ± 2.95, BMI: NR; CG: n = 10, Sex: F, TB: 3 ± 0.8yr., Age: 14 ± 1.8yr, WT: 42 ± 3.4, ht.: 148 ± 3.11, BMI: NR	Freq.: 3 times/week, time: 60 min, Length: 10weeks	Functional strength training group (EG1), control group (CG)	Balance (SST, dynamic balance), strength (handgrip, LS, BS)	SST↑, Dynamic balance↑, Handgrip Strength↔, LS↔, BS↑
Alonso-Fernández et al. (2017)	Pre-post test	Handball players	Sex: F, TB≥ 60 months., Age: 15.2 ± 0.6yr., EG1 = 7, WT: 63.17 ± 9.44, ht.: 164 ± 5, BMI: 23.83 ± 3.46, CG = 7, WT:67.29 ± 0.03, ht.: 166 ± 9.24, BMI: 24.61 ± 3.93,	Freq.: 2 times/week, time: 10 min, Length: 8 weeks	Combining strength, coordination and plyometric exercises (EG1), Control group (CG)	Body composition (WT, BMI, BF%,), VO2max), speed (RSA), power (CMJ: AT, JH, GCT, PEAKPWR)	WT↔, BMI↔, BF%↑, AT↔, JH↔, GCT↔, PEAKPWR↔, RSA↑,
Yildiz et al. (2019)	Pre-mid-post test	Prepubertal tennis players	EG1 = 10, EG2 = 10, CG = 8, Sex: NR, TB: 3.1 ± 1.1yr., Age: 9.6 ± 0.7yr., WT: 31.3 ± 4.1, ht.: 134.1 ± 6.8, BMI: NR	Freq.: 3 times/week, time: 65-70 min, Length: 8 weeks	Functional training group (EG1), Traditional training group (EG2), Control group (CG)	Flexibility (Sit and reach), power (CMJ), speed (10 m test), agility (T-test), balance (RDB, LDB, SB)	Sit and reach↑, CMJ↑, 10 m test↑, T-test↑, RDB↑, LDB↑, SB↑
Baron et al. (2020)	Pre-post test	Young football players	EG1: n = 20, Sex: NR, TB: NR, Age: 16.8 ± 0.6 yr., WT: 66.5 ± 7.4 kg, ht.: 175.7 ± 6.4 cm, BMI: 21.5 ± 1.8	Freq.: NR, time: 70-90 min, Length: 12 weeks	Functional training (EG1)	Speed and acceleration (0-5 m, 5– 10 m, 10–30 m, 30 m)	0-5 m ↔, 5–10 m↑, 10–30 m↑, 30 m↑
Keiner et al. (2020)	Pre-post test	Elite adolescent soccer players	EG1: n = 11, EG2: n = 11, EG3: n = 14. CG: n = 12 Sex: NR, TB: NR, Age: 17.45 ± 0.52yr, WT: 73.0 ± 7.0 kg, ht.: 1.78 ± 0.06 m, BMI: NR	Freq.: 2 times/week, time: 60 min, Length: 10 months	Traditional strength training (EG1), plyometrics and sprint training (EG2), functional training group (EG3), control group (CG)	Speed (20 m, CDS: CODSR, CODSL), power (SJ: AT, JH), strength(1RM)	20 m↑, CDS (CODSR, CODSL)↑, SJ↑, 1RM↑

*↑, significant within-group improvement from pretest to post-test; ↔, non-significant within-group change from pretest to post-test; WT, weight; ht, height; M, Male; yr, year; NR, not reported; Freq., frequency; CG, control group; EG, experimental group; HEX, hexagon test; 5–10–5 test, 5–10–5 meter shuttle run test; SMB, standing overarm medicine ball throw; LMB, lying medicine ball throw; SST, Static strength test; OMBT, overhead medicine ball throw; CMJ, vertical counter movement jump; AT, air time; JH, jump height; GCT: ground contact time; PEAKPWR, power peak; NWP, regular soccer warm-up; WPS, warm-up program supplemented with functional exercises; CDS, change of direction sprint; HIIT, high-intensity interval training; RSA, repeated sprint ability; BM, body mass; BF%, body fat mass percentage; BF, body fat; LBM, lean body mass; H2o(I), total body water; RDB, right dynamic balance; LDB, left dynamic balance; SB, static balance; FJ, ten-fold jump; SQ, Squat for 30s; PU, Push-ups, TLPP, Torso lifting from a prone position for 30s, SJ, Jump performance; 1RM, 1 repetition maximum (squat maximum strength performance); SHR, Shuttle run; LS, leg strength; BS, back strength*.

### Study Selection

**Figure 1:**
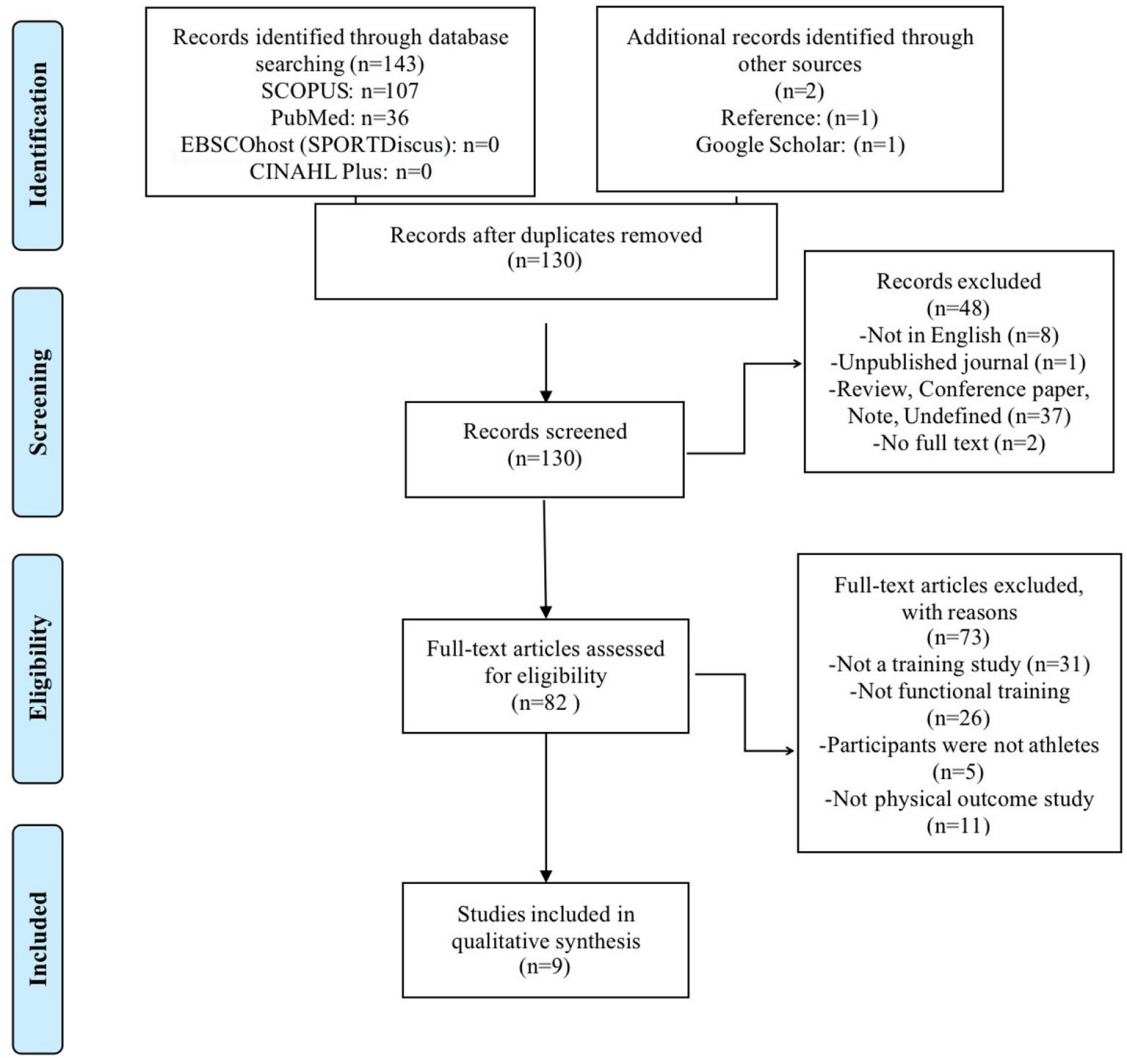
PRISMA flow chart of the study selection process.

Figure 1 shows the flow chart of records selection. A total of 143 potential articles were identified through the electronic database search (36 from PubMed; 107 from SCOPUS; 0 from EBSCOhost (SPORTDiscus); 0 from CINAHL Plus), and additional relevant articles in screening the reference lists of studies that were included in the review and reference lists of previous related reviews (*n* = 1), and Google Scholar (*n* = 1). After exclusion of the duplicates (15), the title and abstract of 130 were assessed for eligibility. After elimination at the title and abstract level 48 articles, the remaining 82 articles were subsequently read. After reading, another 73 articles were eliminated, leaving nine relevant articles that satisfied the inclusion criteria and were included in the qualitative synthesis.

### Study Quality Assessment

An assessment of the study quality, according to the PEDro list, is presented in Table 3. The mean PEDro score of the included studies was 3.44 (range 3–4), which indicates that the included studies were of fair quality, and none of the studies met all the PEDro list quality criteria. All studies specified their eligibility criteria, similar baseline group, between-group comparisons, point measure and variability. None of the studies reported on allocation concealment, blind subject, blind therapist, blind assessor, or intention to treat analysis, except for three studies which described random allocation (Tomljanović et al., 2011; Alonso-Fernández et al., 2017; Yildiz et al., 2019), and only one study reported follow-ups (Oliver and Brezzo, 2009). Nevertheless, it is challenging to include blind subjects, blind therapists, and blind assessors as participants and assessors, since the included studies were exercise training interventions. This situation calls for higher quality and better evidence level studies to be conducted in the future.

**Table 3:** Summary of methodological quality assessment scores.

**References**	**Oliver and Brezzo (2009)**	**Tomljanović et al. (2011)**	**Sander et al. (2013)**	**Elbadry (2014)**	**Cherepov and Shaikhetdinov (2016)**	**Alonso-Fernández et al. (2017)**	**Yildiz et al. (2019)**	**Baron et al. (2020)**	**Keiner et al. (2020)**
Eligibility criteria	1	1	1	1	1	1	1	1	1
Random allocation	0	1	0	0	0	1	1	0	0
Allocation concealment	0	0	0	0	0	0	0	0	0
Group similar at baseline	1	1	1	1	1	1	1	1	1
Blind therapist	0	0	0	0	0	0	0	0	0
Blind assessor	0	0	0	0	0	0	0	0	0
Follow-up	1	0	0	0	0	0	0	0	0
Intention to treat analysis	0	0	0	0	0	0	0	0	0
Between group comparisons	1	1	1	1	1	1	1	1	1
Point measure and variability	1	1	1	1	1	1	1	1	1
PEDro score	4	4	3	3	3	4	4	3	3

### Population Characteristics

The nine included studies' population characteristics were reported based on the following aspects: (1) Athlete classification. In the included literature, only one article did not report the athlete classification (Tomljanović et al., 2011) but only reported moderately trained athletes, and eight articles reported the type of athlete, including football players (Oliver and Brezzo, 2009; Sander et al., 2013; Baron et al., 2020; Keiner et al., 2020), martial artists (Cherepov and Shaikhetdinov, 2016), handball players (Elbadry, 2014; Alonso-Fernández et al., 2017), tennis players (Yildiz et al., 2019) and volleyball players (Oliver and Brezzo, 2009); (2) Sample size. In total, the nine studies consisted of 330 subjects, ranging from 14 (Alonso-Fernández et al., 2017) to 121 (Sander et al., 2013) participants, with a median of 26 (Oliver and Brezzo, 2009) and mean of 36.7; (3) Gender. All nine studies focused on athletes, three studies focused on females (Oliver and Brezzo, 2009; Elbadry, 2014; Alonso-Fernández et al., 2017), one study focused on male (Tomljanović et al., 2011), and the remaining five studies did not report gender (Sander et al., 2013; Cherepov and Shaikhetdinov, 2016; Yildiz et al., 2019; Baron et al., 2020; Keiner et al., 2020); (4) Age. Most studies report the subjects' age, except for one (Cherepov and Shaikhetdinov, 2016), and only one study reported the age range of the subjects (Tomljanović et al., 2011). An analysis of age reports in seven studies found that the age range of the subjects ranged from 9.6 years to 25 years (Oliver and Brezzo, 2009; Sander et al., 2013; Elbadry, 2014; Alonso-Fernández et al., 2017; Yildiz et al., 2019; Baron et al., 2020; Keiner et al., 2020); (5) Body Mass Index. Most studies reported the height and weight of the subjects (Oliver and Brezzo, 2009; Tomljanović et al., 2011; Sander et al., 2013; Elbadry, 2014; Alonso-Fernández et al., 2017; Yildiz et al., 2019; Keiner et al., 2020), only two studies reported the BMI of the subjects (Alonso-Fernández et al., 2017; Baron et al., 2020), and only one study did not state the weight, height, BMI of the subjects (Cherepov and Shaikhetdinov, 2016). For the consistency of literature analysis, the following formula was used to calculate the BMI of the subjects in the relevant studies: BMI = weight (kg)/height^2^ (m). The BMI of the participants in the study ranged from 17.26 to 24.4 kg/m^2^; (6) Training background. Among the nine studies, five studies reported the training background of athletes (Tomljanović et al., 2011; Sander et al., 2013; Elbadry, 2014; Alonso-Fernández et al., 2017; Yildiz et al., 2019) while the other four studies did not describe the training background (Oliver and Brezzo, 2009; Cherepov and Shaikhetdinov, 2016; Baron et al., 2020; Keiner et al., 2020). For the consistency of literature analysis, the training background of the athletes was recorded in months. The training background of the subjects ranged from 36 months to 146 months.

### Interventions Characteristics

The nine included studies' intervention characteristics were reported based on the following aspects: (1) Training length. The shortest intervention length is 8 days (Sander et al., 2013) and the longest being 10 months (Keiner et al., 2020); (2) Duration of each training session. Most studies reported the duration of each training session (Oliver and Brezzo, 2009; Elbadry, 2014; Alonso-Fernández et al., 2017; Yildiz et al., 2019; Baron et al., 2020; Keiner et al., 2020), only three studies did not state the duration (Tomljanović et al., 2011; Sander et al., 2013; Cherepov and Shaikhetdinov, 2016). The duration of each training session analysis of 6 research reports found that they ranged from 10 min (Oliver and Brezzo, 2009; Alonso-Fernández et al., 2017) to 90 min (Baron et al., 2020); (3) Training frequency. Among the nine studies included, six studies reported frequency of training (Oliver and Brezzo, 2009; Tomljanović et al., 2011; Elbadry, 2014; Alonso-Fernández et al., 2017; Yildiz et al., 2019; Keiner et al., 2020) while the other three studies did not (Sander et al., 2013; Cherepov and Shaikhetdinov, 2016; Baron et al., 2020). The frequency analysis of 6 research reports found that the frequency ranged from 2 times/week to 4 times/week (Oliver and Brezzo, 2009; Tomljanović et al., 2011; Elbadry, 2014; Alonso-Fernández et al., 2017; Yildiz et al., 2019; Keiner et al., 2020).

### Outcome and Measures

The outcomes for the present study were grouped according to the effects of functional training on different physical fitness components among athletes. All authors of this study independently classified the papers according to other research topics (components). Disagreements were resolved through discussion among all authors until a consensus was reached.

### Effect of Functional Training on Speed

Six of the nine studies included in this systematic review presented inferences about the effect of functional training on speed performance (Tomljanović et al., 2011; Sander et al., 2013; Alonso-Fernández et al., 2017; Yildiz et al., 2019; Baron et al., 2020; Keiner et al., 2020). The speed tests used in these studies included linear sprint test of 5 m (Sander et al., 2013), 10 m (Tomljanović et al., 2011; Sander et al., 2013; Yildiz et al., 2019; Baron et al., 2020), 15 m (Sander et al., 2013), 20 m (Tomljanović et al., 2011; Sander et al., 2013; Keiner et al., 2020), 25 m (Sander et al., 2013) and 30 m (Sander et al., 2013). They also included change of direction sprint test (5 m left and right test, 10 m left and right test) (Sander et al., 2013; Keiner et al., 2020), repeated sprint ability test (Alonso-Fernández et al., 2017), and acceleration test (0–5 m, 5–10 m, 10–20 m, 10–30 m, 0–30 m) (Tomljanović et al., 2011; Baron et al., 2020). The subjects included young football players (Sander et al., 2013; Baron et al., 2020; Keiner et al., 2020), moderately trained athletes (Tomljanović et al., 2011), handball female players (Alonso-Fernández et al., 2017) and prepubertal tennis players (Yildiz et al., 2019). Four studies showed improvement in linear sprint test (Sander et al., 2013; Yildiz et al., 2019; Keiner et al., 2020), change of direction sprint test (Sander et al., 2013; Keiner et al., 2020) and repeated sprint ability test (Alonso-Fernández et al., 2017). Additionally, Baron et al. (2020) observed a significant improvement for 5–10 m test and 10–30 m test, contrary to the 0–5 m test and 0–30 m test. However, only one study did not observe any significant change in 10, 20, and 10–20 m tests (Tomljanović et al., 2011).

### Effect of Functional Training on Muscular Strength

Muscular strength was only evaluated in five of the studies that were included in this review (Oliver and Brezzo, 2009; Tomljanović et al., 2011; Elbadry, 2014; Cherepov and Shaikhetdinov, 2016; Keiner et al., 2020). The aspects valued and assessment tools used were pull up (Cherepov and Shaikhetdinov, 2016), medicine ball throwing (Tomljanović et al., 2011; Cherepov and Shaikhetdinov, 2016), pronequadra-ped core test, single-leg squat test (Oliver and Brezzo, 2009), 1 repetition maximum (Keiner et al., 2020), handgrip strength test and static strength test (Elbadry, 2014). The subjects include martial artists (Cherepov and Shaikhetdinov, 2016), moderately trained athletes (Tomljanović et al., 2011), collegiate women athletes (volleyball and soccer players) (Oliver and Brezzo, 2009), elite adolescent soccer players (Keiner et al., 2020) and young handball players (Elbadry, 2014). Studies conducted by Cherepov and Shaikhetdinov (2016) and Keiner et al. (2020) revealed a significant increase in muscular strength after the functional training intervention. Nonetheless, three studies observed a significant improvement on standing overarm medicine ball throw (Tomljanović et al., 2011), leg squat (right and left) (Oliver and Brezzo, 2009), back strength test (Elbadry, 2014), but no significant change on lying medicine ball throw (Tomljanović et al., 2011), quadra-ped left and right test (Oliver and Brezzo, 2009), leg strength test and handgrip strength test (Elbadry, 2014).

### Effect of Functional Training on Power

Among the nine studies included in this review, four studies reported on power (Tomljanović et al., 2011; Alonso-Fernández et al., 2017; Yildiz et al., 2019; Keiner et al., 2020), and five studies did not report on it (Oliver and Brezzo, 2009; Sander et al., 2013; Elbadry, 2014; Cherepov and Shaikhetdinov, 2016; Baron et al., 2020). The aspects valued and assessment tools used involved vertical countermovement jump test (jump height, air time, ground contact time, power peak) (Tomljanović et al., 2011; Alonso-Fernández et al., 2017; Yildiz et al., 2019; Keiner et al., 2020). The subjects include moderately trained athletes (Tomljanović et al., 2011), female handball players (Alonso-Fernández et al., 2017), prepubertal tennis players (Yildiz et al., 2019) and elite adolescent soccer players (Keiner et al., 2020). One study had an 8-week intervention period (Yildiz et al., 2019), while the other study had an intervention period of 10 months (Keiner et al., 2020). The results of these studies reveal that functional training can improve power (Yildiz et al., 2019; Keiner et al., 2020). On the other hand, Tomljanović et al. (2011) observed a significant improvement in the countermovement jump test (jump height, ground contact time, power peak) but not for the vertical counter movement jump test (air time) (Tomljanović et al., 2011). However, only one study reported that the 8-week functional training did not yield significant results in the vertical counter movement jump test (flight time, jump height, ground contact time, and power peak) (Alonso-Fernández et al., 2017).

### Effect of Functional Training on Balance

Balance (static and dynamic balance) was valued only in three of the nine studies included in this review. The measurement tools were the dynamic balance (right and left), static balance (Yildiz et al., 2019), biodex balance test (left and right) (Oliver and Brezzo, 2009) and standing stork test (Elbadry, 2014). The subjects include prepubertal tennis players (Yildiz et al., 2019), collegiate women athletes (volleyball and soccer players) (Oliver and Brezzo, 2009), and young handball players (Elbadry, 2014). One study reported an 8-week intervention period (Yildiz et al., 2019), while the other studies had an intervention period of 10 weeks (Elbadry, 2014). The results of these studies reveal that functional training can improve balance (Elbadry, 2014; Yildiz et al., 2019). Nevertheless, one study reported that 13 weeks of functional training did not significantly improve balance (Oliver and Brezzo, 2009).

### Effect of Functional Training on Body Composition

The body composition aspect appeared to be undervalued by the studies included in this review. Only three of the studies assessed this aspect by using different measurement tools like body weight (Oliver and Brezzo, 2009; Tomljanović et al., 2011; Alonso-Fernández et al., 2017), body height (Tomljanović et al., 2011), body fat mass percentage (Tomljanović et al., 2011), lean body mass (Tomljanović et al., 2011), total body water (Tomljanović et al., 2011), body mass index (Oliver and Brezzo, 2009; Alonso-Fernández et al., 2017) and body fat (Oliver and Brezzo, 2009; Alonso-Fernández et al., 2017). The subjects include moderately trained athletes (Tomljanović et al., 2011), handball players (Alonso-Fernández et al., 2017) and collegiate women athletes (volleyball and soccer players) (Oliver and Brezzo, 2009). Two studies observed no significant effect of functional training on body composition (Oliver and Brezzo, 2009; Tomljanović et al., 2011). However, Alonso-Fernández et al. (2017) observed a significant improvement in body fat, but not for body weight and body mass index (Alonso-Fernández et al., 2017).

### Effect of Functional on Agility

Agility was not the main aspect in many of the studies included in this review. Only three studies valued this criterion through four exercises: shuttle run 3 × 10 m (Cherepov and Shaikhetdinov, 2016), agility 5–10–5 test, hexagon test (Tomljanović et al., 2011) and *T*-test (Yildiz et al., 2019). The subjects include martial artists (Cherepov and Shaikhetdinov, 2016), moderately trained athletes (Tomljanović et al., 2011) and prepubertal tennis players (Yildiz et al., 2019). Studies conducted by Cherepov and Shaikhetdinov (2016) and Yildiz et al. (2019) revealed significant increases in agility after the functional training intervention. In contrast, Tomljanović et al. (2011) observed a significant improvement in the hexagon test but no significant change on the agility 5–10–5 test.

### Effect of Functional Training on Flexibility

Only one study included in this systematic review presented inferences about the effect of functional training on flexibility. The flexibility was measured based on the sit and reach test, commonly used in health-related and physical fitness test batteries to evaluate the hamstring and lower back flexibility (Hui and Yuen, 2000). The participants of this study were pre-pubertal tennis players. This study revealed a highly significant improvement in flexibility after 8 weeks of functional training (Yildiz et al., 2019).

### Effect of Functional Training on Muscular Endurance

Muscular endurance was assessed in one out of the nine studies included in this review (Oliver and Brezzo, 2009). This study uses the one-minute sit up test to evaluate muscular endurance (Oliver and Brezzo, 2009). The study subjects included female collegiate volleyball and soccer players. Oliver and Brezzo (2009) reported positive results in this aspect after the intervention.

## Discussion

This systematic review provides a comprehensive overview of the impact of functional training physical fitness among athletes and their bey relevant knowledge for athletes to improve their physical fitness. This revision is intended to be different from other published studies on using the functional training intervention among athletes. The main findings indicated that functional training could increase physical fitness (speed, strength, power, flexibility, agility, balance, aerobic, and muscular endurance) among athletes. However, no data was found in reaction time and coordination reporting. The reviewed papers varied significantly regarding the participants (type of athletes, age, and gender) and the physical fitness components studied. Nonetheless, functional training may be an effective physical fitness intervention among athletes based on positive findings in these studies. Following the framework in the “Results” section, the physical fitness components of the studies were analyzed in detail.

### Effect of Functional Training on Speed

Most sports experts agree that speed, an elementary motor skill, is vital to succeeding in many sports disciplines (Karalejić et al., 2014). Six studies evaluated this component in their research by using the linear sprint test (Sander et al., 2013; Yildiz et al., 2019; Keiner et al., 2020), change of direction sprint test (Sander et al., 2013; Keiner et al., 2020), and repeated sprint ability test (Alonso-Fernández et al., 2017), which yielded significantly positive results. However, one study reported that some of the measures of repeated sprint ability test (5–10 m, 10–30 m) exhibited a significant increase, but there was no significant change on repeated sprint ability test (0–5 m, 0–30 m) after functional training (Baron et al., 2020). Furthermore, only one study did not observe a significant effect of functional training on linear sprint test (10, 20, 10–20 m test) (Tomljanović et al., 2011). This finding may be a result of improvements in functional status and increased speed. Campa et al. (2019) also demonstrated that better movement patterns might improve speed performance. However, wrong movement patterns will negatively impact the ability to perform fundamental movement patterns with precision and appropriate efficiency, besides increasing the risk of athletic injuries (Kollock et al., 2019).

### Effect of Functional Training on Muscular Strength

Strength has a great influence on physical fitness components. Athletes must intensify strength training to improve their skills and maintain a good competitive state in their respective fields (Dengguang and Yang, 2007b). Meanwhile, muscle strength can be divided into upper limb muscle strength study (Tomljanović et al., 2011; Elbadry, 2014; Cherepov and Shaikhetdinov, 2016) and lower limb muscle strength study (Oliver and Brezzo, 2009; Elbadry, 2014; Keiner et al., 2020). Three studies reported on upper limb strength; one study reported significant improvement in muscular strength (pull up, 3 kg stuffed ball overhead throwing) (Cherepov and Shaikhetdinov, 2016) while the other study reported no significant increases in static strength test (handgrip strength and back strength test) (Elbadry, 2014). However, only one study reported that some of the measures of upper limb muscular strength have significant increases (standing overarm medicine ball throw), but there was no significant improvement in the muscular strength test (lying medicine ball throw) (Tomljanović et al., 2011).

Regarding those studies that assessed muscle strength of lower limbs, one study reported significant improvement in muscular strength (1 RM) (Keiner et al., 2020). On the contrary, no significant improvement in muscular strength (static strength test: leg strength test) was found in another study (Elbadry, 2014). Furthermore, one study reported that some of the measures of lower limb muscular strength (left leg squat and right leg squat) showed significant improvements, but the findings were not statistically significant (quadra-ped left and right test) (Oliver and Brezzo, 2009). In terms of upper limb muscular strength, better results are observed in studies with long-term interventions (Cherepov and Shaikhetdinov, 2016; Keiner et al., 2020). In addition, only one of the five included studies on muscle strength reported on upper limb muscular strength and lower limb muscular strength (Elbadry, 2014). Therefore, more research should analyze strength.

### Effect of Functional Training on Power

Based on the findings of four studies that analyze the benefits of functional training on power, it was not possible to draw a definite conclusion on this aspect. Two studies confirmed that functional training programs increase athletes' performances in the vertical countermovement jump test (Yildiz et al., 2019; Keiner et al., 2020). However, these two studies alone are not enough to support the idea that functional training is beneficial in improving athletes' power, and it is worth noting that this hypothesis has been supported by literature from non-athlete participants (Miszko et al., 2003; Liu et al., 2014).

In addition, Tomljanović et al. (2011) reported that some of the measures of vertical countermovement jump test (jump height, ground contact time, power peak) were statistically significant, but there was no significant increase in the vertical countermovement jump test (air time) (Tomljanović et al., 2011). The functional training program consisted of mostly upper body exercises, which did not test the performance of the lower body. This improvement may be mainly related to neural coordination of movement (Tomljanović et al., 2011). However, another study reported that high-intensity interval training protocols based on functional exercises program had no significant increases in power (flight time, jump height, jump speed) (Alonso-Fernández et al., 2017). This finding is consistent with the studies by Buchheit et al. (2009) and Rey et al. (Viaño-Santasmarinas et al., 2018). However, these data opposed the reported results by Dello Iacono et al. (2016) involving handball players because the functional training program might not be enough to stimulate the neuromuscular system related to power (Luo et al., 2005). Power in the upper/lower extremities is necessary to produce explosive actions among athletes (Girard and Millet, 2009; Chelly et al., 2010). However, the included study only reported the effect of functional training on lower body power but ignored the effect of functional training on upper body power, which was an important gap in the existing literature. Therefore, it is necessary to consider and correct the research on power in functional training.

### Effect of Functional Training on Balance

The static and dynamic balance were tested in three of the nine studies included in this review. Two studies confirmed that a functional training program increases static balance and dynamic balance (Elbadry, 2014; Yildiz et al., 2019). This finding may be explained by the adaptations that occurred in all the sensory systems assisting postural control, such as the vestibular, visual, and the somatosensory and motor systems controlling muscular output (Taube et al., 2008; Latorre Román et al., 2015).

However, only one study conducted was observed that performance in the biodex balance test (left, right) was not significantly improved (Oliver and Brezzo, 2009). This observation may result from all the subjects being in-season, not only in practice and competition, but also in a regimen strength and conditioning program (Oliver and Brezzo, 2009). Therefore, the interference of other factors (e.g., exercise training factors) should be avoided in future research.

### Effect of Functional Training on Body Composition

Three studies showed no significant effect of functional training on body weight (Oliver and Brezzo, 2009; Tomljanović et al., 2011; Alonso-Fernández et al., 2017), body height (Tomljanović et al., 2011), body mass index (Oliver and Brezzo, 2009; Tomljanović et al., 2011; Alonso-Fernández et al., 2017), lean body mass (Tomljanović et al., 2011), and total body water (Tomljanović et al., 2011). The study by Alonso-Fernández et al. (2017) reported statistically significant improvement in body fat, whereas two other studies showed no statistical significance in terms of body composition (Oliver and Brezzo, 2009; Tomljanović et al., 2011). These results are in line with those obtained by Camacho-cardenosa et al. (2016) who found no statistically significant reduction in body fat. Considering that calorie intake and food monitoring have a statistically significant impact on this variable, it is safe to assume that athletes with more regular and stable eating habits may enhance body composition quality (Mettler et al., 2010).

### Effect of Functional Training on Agility

Agility is an essential component in most field and team sports. Traditional definitions of agility have simply identified speed in directional changes as the defining component (Draper and Lancaster, 1985). Out of the three studies that investigated agility (Tomljanović et al., 2011; Cherepov and Shaikhetdinov, 2016; Yildiz et al., 2019), only one of them reported that the agility 5-10-5 test did not demonstrate significant improvement (Tomljanović et al., 2011). The explanation of these results may be the improved power qualities and enhanced postural control of the subjects (Marković et al., 2007). In contrast, the impact of power and explosive strength is lower in speed-led agility tests (e.g., agility 5–10–5) (Tomljanović et al., 2011).

Furthermore, in a study that compared the functional movement screen training and traditional training on agility in 62 elite male high school baseball players, the training program included static stretching, and it was showed that functional movement screen program improves flexibility (trunk flexion forward, trunk extension backward, the splits) (Song et al., 2014). Despite the findings reported in previous studies, functional training may be an effective way to increase agility. Future research should consider exercise items in functional training and only determine the effect of functional training on speed-led agility tests.

### Effect of Functional Training on Flexibility

The sit and reach is a field test used to assess hamstring and lower back flexibility (Baltaci et al., 2003). This study found that the functional training group showed significant improvement at sit and reach, whereas no significant improvements were observed in the traditional training and control groups (Yildiz et al., 2019). Similarly, Weiss et al. reported that the 7 weeks functional training program intervention resulted in significant improvements in the flexibility of college students (non-athletes) (Weiss et al., 2010), which is different from the participants included in the study. However, the functional training intervention can significantly improve the flexibility of the participants. Therefore, these results can only be regarded as weak evidence at present, and they need to be compared to more exercise training interventions.

### Effect of Functional Training on Muscular Endurance

Muscular endurance was measured with a one-minute sit up test (Pritchard et al., 2001). In the studies, the no intervention group also showed significant improvement on the one-minute sit up test. The intervention group's significant improvement and the non-intervention group may be due to the routine training program during the season (Oliver and Brezzo, 2009). However, the sit up test measures rectus abdominal endurance and not deep core musculature (Diener, 1995), which may be why the intervention group and non-intervention group did not show significant improvement.

## Limitations

Overall, this review provides substantial evidence of fair quality and the beneficial effects of different functional training programs on physical fitness among athletes. However, there are several limitations to this review. Firstly, only four studies reported the gender of athletes (Oliver and Brezzo, 2009; Tomljanović et al., 2011; Elbadry, 2014; Alonso-Fernández et al., 2017). If present, it could be important, as there are differences in assessing physical fitness components based on gender. This may impact the final research results. Secondly, none of the studies included in this review stated the sample size calculation method. Determining the sample size is influenced by several factors, including the purpose of the study, population size, the risk of selecting a “bad” sample, and the allowable sampling error (MacCallum et al., 1999). Thus, inappropriate, inadequate, or excessive sample sizes can influence quality and accuracy (Rodríguez del Águila and González-Ramírez, 2014). If the sample size calculation method in the included research is wrong, it may influence the outcome of the study. Thirdly, most studies did not document or control exercises that were performed by participants outside of the study setting. Additionally, most studies did not consider the influence of temperature, time, and other factors on physical fitness among athletes. Finally, the studies did not have any short-term or long-term follow-up, making it difficult to predict the long-term impact of functional training on physical fitness among athletes.

## Conclusion

The present analysis of this systematic review provides strong evidence that functional training improved physical fitness in terms of speed, muscular strength, power, balance, and agility, while there is moderate evidence of the effect on flexibility and muscular endurance. No significant improvement was found in body composition. The results support the principle of specificity in training, where the best gains in performance are achieved when the training closely mimics the performance (Hawley, 2008; Reilly et al., 2009). Furthermore, functional training is a relatively new training modality, but it recently has gained momentum among physical fitness training and has been identified as a “Top 10 Fitness Trend” in 2018 (Thompson, 2017), with four of the nine studies being published in the past 3 years. Moreover, review trials show that functional training was most common in resistance and strength training. Nevertheless, it is necessary to be cautious about the results in view of the limitations outlined in the present study. To better understand the effectiveness of functional training in improving athletes' physical fitness, additional research to examine the effect of functional training on physical fitness components according to the difference in the type of athletes is encouraged. It will help verify the effectiveness of functional training to improve the physical fitness components among different types of athletes and promote functional training in the field of modern sports science (Osipov et al., 2017).

## Data Availability Statement

The original contributions presented in the study are included in the article/supplementary material„ further inquiries can be directed to the corresponding author.

## Author Contributions

The literature search and selection of studies was performed by authors WX and KS. Following an initial screen of titles and abstracts WX, full scrutiny of potentially eligible studies was independently screened by WX and KS using the specific inclusion criteria. OT arbitrated any disagreements in study inclusion. Study quality assessment was performed by WX. All authors contributed to manuscript revision, read, and approved the submitted version.

## Conflict of Interest

The authors declare that the research was conducted in the absence of any commercial or financial relationships that could be construed as a potential conflict of interest.

## Publisher's Note

All claims expressed in this article are solely those of the authors and do not necessarily represent those of their affiliated organizations, or those of the publisher, the editors and the reviewers. Any product that may be evaluated in this article, or claim that may be made by its manufacturer, is not guaranteed or endorsed by the publisher.

## References

[B1] Alexander N. B. Galecki A. T. Grenier M. L. Nyquist L. V. Hofmeyer M. R. Grunawalt J. C.. (2001). Task-specific resistance training to improve the ability of activities of daily living-impaired older adults to rise from a bed and from a chair. J. Am. Geriatr. Soc. 49, 1418–1427. 10.1046/j.1532-5415.2001.4911232.x 11890578

[B2] Alonso-Fernández D. Lima-Correa F. Gutierrez-Sánchez F. De Vicuña O. A. G. (2017). Effects of a high-intensity interval training protocol based on functional exercises on performance and body composition in handball female players. J. Hum. Sport Exerc. 12, 1186–1198. 10.14198/jhse.2017.124.05

[B3] Baltaci G. Un N. Tunay V. Besler A. Gerçeker S. (2003). Comparison of three different sit and reach tests for measurement of hamstring flexibility in female University students. Br. J. Sports Med. 37, 59–61. 10.1136/bjsm.37.1.59 12547745 PMC1724584

[B4] Baron J. Bieniec A. Swinarew A. S. Gabryś T. Stanula A. (2020). Effect of 12-week functional training intervention on the speed of young footballers. Int. J. Environ. Res. Public Health 17, 1–11. 10.3390/ijerph17010160 31878326 PMC6981857

[B5] Boyle M. (2016). New Functional Training for Sports. Human Kinetics.

[B6] Buchheit M. Laursen P. B. Kuhnle J. Ruch D. Renaud C. Ahmaidi S. (2009). Game-based training in young elite handball players. Int. J. Sports Med. 30:251. 10.1055/s-0028-1105943 19199207

[B7] Camacho-cardenosaA.Brazo-sayaveraJ.Camacho-cardenosaM.Marcos,-M.TimónR.CienciasF.. (2016). Effects of high intensity interval training on fat mass parameters in adolescents. Rev. Esp. Salud Publica90, 1–9. 10.1055/VOL90/ORIGINALES/RS90C_ACC.pdf27869113

[B8] Campa F. Semprini G. Júdice P. B. Messina G. Toselli S. (2019). Anthropometry, physical and movement features, and repeated-sprint ability in soccer players. Int. J. Sports Med. 40, 100–109. 10.1055/a-0781-2473 30557888

[B9] Chelly M. S. Hermassi S. Shephard R. J. (2010). Relationships between power and strength of the upper and lower limb muscles and throwing velocity in male handball players. J. Strength Cond. Res. 24, 1480–1487. 10.1519/JSC.0b013e3181d32fbf 20508448

[B10] Cherepov E. A. Shaikhetdinov R. G. (2016). Effectiveness of functional training during physical conditioning of students practicing martial arts. J. Phys. Educ. Sport 16, 510–512. 10.7752/jpes.2016.02079

[B11] Chunlei L. (2016). Design and implementation of physical fitness training of China national badminton team in preparing for 2012 London Olympic Games. J. Beijing Sport Univ. 5, 60–69. 10.19582/j.cnki.11-3785/g8.2016.05.015

[B12] Cook G. E. (2012). Movement functional movement systems: screening, assessment and corrective strategies. J. Can. Chiropr. Assoc. 56:158.

[B13] Dello Iacono A. Ardig,ò L. P. Meckel Y. Padulo J. (2016). Effect of small-sided games and repeated shuffle sprint training on physical performance in elite handball players. J. Strength Cond. Res. 30, 830–840. 10.1519/JSC.0000000000001139 26907846

[B14] Dengguang L. Yang Z. (2007a). Physiological characteristics of strength training in the tennis project athletes. J. Jilin Inst. Phys. Educ. 23, 52–53.

[B15] Dengguang L. Yang Z. (2007b). Physiological characteristics of strength training in the tennis project athletes. J. Jilin Inst. Phys. Educ. 6, 52–53.

[B16] Diener D. (1995). Validity and reliability of a one-minute half sit-up test of abdominal strength and endurance. Sport. Med. Train. Rehabil. 6, 105–109. 10.1080/15438629509512042

[B17] Draper J. A. Lancaster M. G. (1985). The 505 test: a test for agility in the horizontal plane. Aust. J. Sci. Med. Sport. 24, 919–925. Available online at: https://www.brianmac.co.uk/agility505.htm

[B18] Elbadry N. (2014). Effect of functional strength training on certain physical variables and performance level of hammer throw. Ovidius Univ. Ann. Ser. Phys. Educ. Sport. Mov. Heal. 26, 495–499. Available online at: https://www.analefefs.ro/anale-fefs/2014/i2_supp/pe-autori/26.pdf

[B19] Feito Y. Heinrich K. Butcher S. Poston W. (2018). High-intensity functional training (HIFT): definition and research implications for improved fitness. Sports 6:76. 10.3390/sports6030076 30087252 PMC6162410

[B20] Fernandez-Fernandez J. Sáez De Villarreal E. Sanz-Rivas D. Moya M. (2016). The effects of 8-week plyometric training on physical performance in young tennis players. Pediatr. Exerc. Sci. 28, 77–86. 10.1123/pes.2015-0019 26252503

[B21] Foley N. C. Teasell R. W. Bhogal S. K. Speechley M. R. (2003). Stroke rehabilitation evidence-based review: methodology. Top. Stroke Rehabil. 10, 1–7. 10.1310/Y6TG-1KQ9-LEDQ-64L8 12970828

[B22] Gabbett T. Kelly J. Pezet T. (2007). Relationship between physical fitness and playing ability in rugby league players. J. Strength Cond. Res. 21, 1126–1133. 10.1519/R-20936.1 18076242

[B23] Giné-Garriga M. Guerra M. Pagès E. Manini T. M. Jiménez R. Unnithan V. B. (2010). The effect of functional circuit training on physical frailty in frail older adults: a randomized controlled trial. J. Aging Phys. Act. 18, 401–424. 10.1123/japa.18.4.401 20956842

[B24] Girard O. Millet G. P. (2009). Physical determinants of tennis performance in competitive teenage players. J. Strength Cond. Res. 23, 1867–1872. 10.1519/JSC.0b013e3181b3df89 19675471

[B25] Hawley J. A. (2008). Specificity of training adaptation: time for a rethink? J. Physiol. 586, 1–2. 10.1113/jphysiol.2007.147397 18167367 PMC2375570

[B26] Hui S. S. C. Yuen P. Y. (2000). Validity of the modified back-saver sit-and-reach test: a comparison with other protocols. Med. Sci. Sports Exerc. 32, 1155–1659. 10.1097/00005768-200009000-00021 10994920

[B27] Karalejić S. Stojiljković D. Stojanović J. Andelković I. Nikolić D. (2014). Methodics of developing speed in young athletes. Act. Phys. Educ. Sport 14, 158–161. Available online at: https://fsprm.mk/wp-content/uploads/2014/11/Pages-from-APES-ZA-NA-EMAIL-16.pdf

[B28] Keiner M. Kadlubowski B. Sander A. Hartmann H. Wirth K. (2020). Effects of 10 months of speed, functional, and traditional strength training on strength, linear sprint, change of direction, and jump performance in trained adolescent soccer players. J. Strength Cond. Res. Publish Ah. 27:3807. 10.1519/JSC.0000000000003807 32868678

[B29] Kollock R. O. Lyons M. Sanders G. Hale D. (2019). The effectiveness of the functional movement screen in determining injury risk in tactical occupations. Ind. Health. 86. 10.2486/indhealth.2018-0086 30393251 PMC6685800

[B30] Kovacs M. S. (2006). Applied physiology of tennis performance. Br. J. Sports Med. 40, 381–386. 10.1136/bjsm.2005.023309 16632565 PMC2653871

[B31] Latorre Román P. Á. Santos E Campos M. A. García-Pinillos F. (2015). Effects of functional training on pain, leg strength, and balance in women with fibromyalgia. Mod. Rheumatol. 25, 943–947. 10.3109/14397595.2015.1040614 25867230

[B32] Li Z. (2021). Research on improving the core strength of tennis players by functional training. J. Anhui Norm. Univ. Sci. 44, 187–191. 10.14182/J.cnki.1001-2443.2021.02.012

[B33] Li Z. Zhao H. Chang Y. (2019). Analysis on the connotation purport,structure function and essential attributes of functional training to strength and conditioning. J. Tianjin Univ. Sport 34, 227–231. 10.13297/j.cnki.issn1005-0000.2019.03.008

[B34] Lima L. O. Scianni A. Rodrigues-de-Paula F. (2013). Progressive resistance exercise improves strength and physical performance in people with mild to moderate Parkinson's disease: a systematic review. J. Physiother. 59, 7–13. 10.1016/S1836-9553(13)70141-3 23419910

[B35] Liu C. Shiroy D. M. Jones L. Y. Clark D. O. (2014). Systematic review of functional training on muscle strength, physical functioning, and activities of daily living in older adults. Eur. Rev. Aging Phys. Act. 11, 95–106. 10.1007/s11556-014-0144-119588334

[B36] Luo J. McNamara B. Moran K. (2005). The use of vibration training to enhance muscle strength and power. Sport. Med. 35, 23–41. 10.2165/00007256-200535010-00003 15651911

[B37] MacCallum R. C. Widaman K. F. Zhang S. Hong S. (1999). Sample size in factor analysis. Psychol. Methods 4:84. 10.1037/1082-989X.4.1.84

[B38] Marković G. Sekulić D. Marković M. (2007). Is agility related to strength qualities? analysis in latent space. Coll. Antropol. 31, 787–793. https://www.researchgate.net/profile/Goran_Markovic3/publication/5807221_Is_agility_related_to_strength_qualities_-_Analysis_in_latent_space/links/02e7e52d1502d0d580000000.pdf 18041390

[B39] Mettler S. Mitchell N. Tipton K. D. (2010). Increased protein intake reduces lean body mass loss during weight loss in athletes. Med. Sci. Sports Exerc. 42, 326–337. 10.1249/MSS.0b013e3181b2ef8e 19927027

[B40] Miszko T. A. Cress M. E. Slade J. M. Covey C. J. Agrawal S. K. Doerr C. E. (2003). Effect of strength and power training on physical function in community-dwelling older adults. J. Gerontol. Ser. A Biol. Sci. Med. Sci. 58, 171–175. 10.1093/gerona/58.2.M171 12586856

[B41] Moher D. Liberati A. Tetzlaff J. Altman D. G. Altman D. Antes G.. (2009). Preferred reporting items for systematic reviews and meta-analyses: the PRISMA statement. PLoS Med. 6:e1000097. 10.1371/journal.pmed.1000097 19621072 PMC2707599

[B42] National Academy of Sports Medicine (2001). *Integrated kinetic chain* assessment, in National Academy of Sports Medicine.

[B43] Oliver G. D. Brezzo R. (2009). Functional balance training in collegiate women athletes. J. Strength Cond. Res. 23, 2124–2129. 10.1519/JSC.0b013e3181b3dd9e 19855341

[B44] Osipov A. Kudryavtsev M. Gatilov K. Zhavner T. Klimuk Y. Ponomareva E.. (2017). The use of functional training – Crossfit methods to improve the level of special training of athletes who specialize in combat sambo. J. Phys. Educ. Sport 17, 2013–2018. 10.7752/jpes.2017.03201

[B45] Pacheco M. M. Teixeira L. A. C. Franchini E. Takito M. Y. (2013). Functional vs. strength training in adults: specific needs define the best intervention. Int. J. Sports Phys. Ther. 8:34. 23439782 PMC3578432

[B46] Pritchard T. O'Bryant H. Johnson R. Everhart B. (2001). An alternative to the full sit-up testing for middle school students. Phys. Educ. 58, 42. Available online at: https://www.proquest.com/docview/232988386?pq-origsite=gscholar&fromopenview=true

[B47] Reilly T. Morris T. Whyte G. (2009). The specificity of training prescription and physiological assessment: a review. J. Sports Sci. 27, 575–589. 10.1080/02640410902729741 19340630

[B48] Rodríguez del Águila M. M. González-Ramírez A. R. (2014). Sample size calculation. Allergol. Immunopathol. 1:55. 10.1016/j.aller.2013.03.008 24280317

[B49] Sander A. Keiner M. Schlumberger A. Wirth K. Schmidtbleicher D. (2013). Effects of functional exercises in the warm-up on sprint performances. J. Strength Cond. Res. 27, 995–1001. 10.1519/JSC.0b013e318260ec5e 22692105

[B50] Santos-rosa F. J. Fernandez-fernandez J. Garc V. Teixeira A. S. (2020). The effect of a neuromuscular vs. dynamic warm-up on physical performance in young tennis players. J. Strength Cond. Res. 34, 2776–2784. 10.1519/JSC.0000000000003703 32986392

[B51] Skelton D. A. Young A. Greig C. A. Malbut K. E. (1995). Effects of resistance training on strength, power, and selected functional abilities of women aged 75 and older. J. Am. Geriatr. Soc. 43, 1081–1087. 10.1111/j.1532-5415.1995.tb07004.x 7560695

[B52] Smith D. J. (2003). A framework for understanding the training process leading to elite performance. Sport. Med. 33, 1103–1126. 10.2165/00007256-200333150-00003 14719980

[B53] Song H. S. Woo S. S. So W. Y. Kim K. J. Lee J. Kim J. Y. (2014). Effects of 16-week functional movement screen training program on strength and flexibility of elite high school baseball players. J. Exerc. Rehabil. 10, 124–130. 10.12965/jer.140101 24877049 PMC4025546

[B54] Taube W. Gruber M. Gollhofer A. (2008). Spinal and supraspinal adaptations associated with balance training and their functional relevance. Acta Physiol. 193, 101–116. 10.1111/j.1748-1716.2008.01850.x 18346210

[B55] Thompson W. R. (2017). Worldwide survey of fitness trends for 2018: The CREP Edition. ACSM's Heal. Fit. J. 21, 10–19. 10.1249/FIT.0000000000000341

[B56] Tomljanović M. Spasić M. Gabrilo G. Uljević O. Foretić N. (2011). Effects of five weeks of functional vs. traditional resistance training on anthropometric and motor performance variables. Kinesiology 43, 145–154. https://www.academia.edu/24832028/Effects_of_five_weeks_of_functional_vs._traditional_resistance_training_on_anthropometric_and_motor_performance_variables

[B57] Viaño-Santasmarinas J. Rey E. Carballeira S. Padrón-Cabo A. (2018). Effects of high-intensity interval training with different interval durations on physical performance in handball players. J. Strength Cond. Res. 32, 3389–3397. 10.1519/JSC.0000000000001847 28195979

[B58] Weiss T. Kreitinger J. Wilde H. Wiora C. Steege M. Dalleck L.. (2010). Effect of functional resistance training on muscular fitness outcomes in young adults. J. Exerc. Sci. Fit. 8, 113–122. 10.1016/S1728-869X(10)60017-2

[B59] Yildiz S. Pinar S. Gelen E. (2019). Effects of 8-week functional vs. traditional training on athletic performance and functional movement on prepubertal tennis players. J. Strength Cond. Res. 33, 651–661. 10.1519/JSC.0000000000002956 30431536

